# Supplementary material for: Are carbon emissions trading and green financial instruments synergistic? -Comprehensive quantitative research based on content analysis
Source: PLoS One. 2024 Mar 7;19(3):e0298601. doi: 10.1371/journal.pone.0298601 (PMC10919601; doi:10.1371/journal.pone.0298601)
Supplement: S1 Data — (ZIP) [file pone.0298601.s001.zip › Data/地级市Z_F工作报告.zip!/▒▒╛⌐/└┤╫╘╣1⁄2╓┌║┼í╛┬φ┐╦╩2╛▌═°í┐_2011.docx]

——2011年1月16日在北京市第十三届人民代表大会第四次会议上

北京市市长 郭金龙

各位代表：

　　现在，我代表北京市人民政府，向大会报告政府工作，并对“十二五”规划《纲要(草案)》作说明，请予审议，请市政协各位委员提出意见。

　　一、2010年工作回顾

　　2010年，面对极为复杂的国内外环境，在党中央、国务院和中共北京市委坚强领导下，全市人民深入贯彻落实科学发展观，着眼建设中国特色世界城市，全面实施人文北京、科技北京、绿色北京战略，加快转变经济发展方式，巩固应对国际金融危机成果，推动首都经济社会发展取得了新的显著成绩。初步预计，全市地区生产总值比上年增长10%以上；地方财政一般预算收入增长16.1%；年末城镇登记失业率为1.37%；城镇居民人均可支配收入、农村居民人均纯收入分别增长8.5%以上和10.5%以上，扣除价格因素，分别实际增长6%以上和8%以上。“十一五”规划的主要目标任务胜利完成，首都改革开放和现代化建设迈出了新的坚实步伐。

　　一年来，我们认真落实市十三届人大三次会议决议，紧紧围绕推动首都科学发展，主要做了以下几方面工作：

　　(一)全力保持经济平稳较快发展，夯实可持续发展的基础

　　坚持把扩大消费放在突出位置，全面落实国家各项政策措施，着力优化消费环境，完善商品流通服务体系，组织开展了系列主题促销活动，大力推进农超对接、农村集贸市场建设等工作，预计全年社会消费品零售额超过6200亿元、增长17%以上。实行稳物价、保供应的一系列措施，居民消费价格涨幅控制在3%以内。建立政府资金统筹保障机制，加快推进重大项目和重点区域建设，完成土地储备开发投资1470亿元。积极调整投资结构，工业、第三产业投资分别增长28%以上和12%以上，民间投资比重接近58%。完成全社会固定资产投资近5500亿元、增长13%以上，超额实现两年政府投资1400亿元、带动社会投资1万亿元的目标。

　　加快中关村国家自主创新示范区建设。发布实施示范区建设三年行动计划和十年规划纲要，国务院同意实行“1+6”系列政策。着力推进先行先试改革，股权激励试点单位达到350家，科技专项经费列支间接费用试点单位161家，政府采购自主创新产品51.5亿元。成立中关村发展集团，建立产业化促进机制，推动60项重大科技成果产业化，示范区规模以上企业预计实现总收入突破1.5万亿元、增长20%。制定首都中长期人才发展规划纲要，全力推进中关村科学城、未来科技城和人才特区建设，实施海外人才聚集、高端领军人才聚集工程，引进了一批高端领军人才和团队。加快创新型城市建设，落实首都知识产权战略，建立研发试验服务合作网络，促进高校科研成果开放共享，做好国家科技重大专项对接服务，协同推进6项国家重大科技基础设施建设，全市技术合同成交额达到1579亿元、增长27.8%。

　　深化产业结构调整。积极推进西部地区转型发展，首钢冶炼热轧全面停产，关闭所有小煤窑。退出“三高”企业43家。制定实施加快物流、信息服务、商务服务、旅游、设计产业发展的政策措施，出台促进首都功能核心区文化发展的意见，生产性服务业、文化创意产业较快增长。旅游总收入达到2768亿元，增长13.3%。规模以上工业企业增加值增长15%左右，实现利润增长40%左右。大力开发农业多种功能，都市型现代农业加快发展。坚持调整产业结构与落实区县功能定位相结合，重点功能区建设全面加强，通州现代化国际新城建设快速推进，产业发展聚集带动作用不断增强。

　　统筹推进城乡区域协调发展。深化农村改革，完善农地流转服务体系，全面推进集体林权制度、集体经济产权制度改革，对山区集体公益林进行补助，农民专业合作社覆盖70%从事一产的农户。大力推进新农村建设，五项基础设施提前两年实现全覆盖。实施城乡结合部城市化工程，完成24个重点村拆迁任务。落实城南行动计划，103项重大基础设施和产业项目进展顺利。加快永定河绿色生态发展带建设，河道景观恢复和生态环境修复初见成效。完成7条重点沟域规划国际招标，建设生态清洁小流域22条，生态涵养发展区建设迈出新步伐。

　　(二)加强城乡建设、服务与管理，不断完善城市功能

　　加快基础设施建设。大力发展轨道交通，5条新线开通试运营，新增通车里程108公里。推进完善中心城区路网系统，3条城市快速路建成通车，完成110项疏堵工程。优化公交线网，公交出行比例提高到40.1%。强化资源能源保障，建成陕京天然气三线，完成第九水厂改造，解决了中心城区6万户居民老楼通气问题，实现230个老旧小区管网供热。实施信息化基础设施提升计划，城市信息化水平不断提高。

　　切实改善生态环境。坚持增能力、调结构、促减量，全面推进生活垃圾处理工作。全市生活垃圾产生量继续下降，日处理能力达到1.7万吨，无害化处理率提高到96%。推进卢沟桥等再生水厂改造升级，全市再生水利用率、污水处理率分别达到60%和81%。完成农民住宅节能改造2万户、城镇建筑节能改造644万平方米、城区平房煤改电1.3万户、燃煤锅炉清洁能源改造1052蒸吨，淘汰黄标车5万辆，新增纯电动公交、环卫示范车1260辆，市区空气质量二级和好于二级天数占78.4%。加强北运河水系综合治理。积极推进新城滨河森林公园、郊野公园建设，新增造林面积16万亩。

　　强化城市服务管理。成立首都城市环境建设委员会、北京历史文化名城保护委员会，完善城六区交通管理体制。着力加强交通管理，实施错时上下班等一揽子措施，维持了城区路网的基本通行能力。按照“建、管、限”的思路，出台缓解交通拥堵的28项综合措施。加强重点地区和薄弱环节环境整治，完成了100公里架空线入地任务。总结推广村庄社区化管理模式，郊区村庄管理水平明显提高。全面加强人口和计划生育工作。第六次人口普查工作顺利推进。

　　创新社会服务管理。出台社会服务管理创新行动方案。开展1000个社区规范化建设试点，建成1294个农村社区服务站。向社会组织购买300个服务项目，选聘2958名大学生到社区工作，较大幅度提高了社区工作者待遇。基本实现商务楼宇服务站全覆盖，启动社区服务管理创新综合试点，推进“一刻钟社区服务圈”示范点建设。新建16支专业化志愿者队伍，志愿者服务管理进一步规范。

　　(三)加强以改善民生为重点的社会建设，维护首都和谐稳定

　　全力做好就业和社会保障工作。强化高校毕业生就业综合服务，完善“零就业家庭”、纯农就业家庭长效帮扶机制，实现城镇新增就业44.6万人、农村劳动力转移就业9.6万人。整合“一老一小”、无业居民大病医疗保险制度，制定统一的城镇居民基本医疗保险办法，区县公费医疗与职工基本医疗保险并轨。实施提高门诊报销比例等8项医保惠民政策，减轻群众负担25亿元。完成社保卡整体工程建设，1779家定点医疗机构实现就医即时结算。出台养老保险补缴政策，实现了养老保险关系转移接续。全面落实“九养政策”和老年优待办法，发放养老助残服务券4.3亿元，为2.1万户家庭实施了无障碍设施改造。

　　不断提升基本公共服务水平。制定中长期教育改革和发展规划纲要。全面实现免费义务教育。加快推进中小学校舍安全工程，累计开工346万平方米。改扩建30所公办幼儿园，增加入园名额1.2万个。延长城区社区卫生服务中心门诊时间，26家三级医院开展双休日全天门诊，启动预约转诊试点。继续实施周末演出场计划等文化惠民工程，基本完成街道、行政村文化中心建设任务，率先实现“村村有书屋”。加强历史文化名城保护，完成29项文物修缮工程。成功举办首届世界武搏会等重大赛事，北京运动员在国内外赛场上取得优异成绩，全民健身运动蓬勃发展。

　　加大住房保障力度。政策性住房实际供地1332公顷，同比增长1倍，占全市住宅用地供应量的52.8%。新建和收购政策性住房22.5万套，占全市住宅新开工套数的61.5%。竣工各类政策性住房5万套，完成年度计划的108.7%。公共租赁住房落实房源2.6万套，廉租住房基本实现了实物配租应保尽保。三区三片棚户区改造累计搬迁居民1.6万户，首都功能核心区居民对接安置房开工218万平方米。严格执行中央房地产调控政策，加强房地产市场监管，5月份以来房屋销售价格同比涨幅逐月回落。

　　高度重视做好安全稳定工作。建立重大事项社会稳定风险评估机制，推广信访代理等成功经验，着力化解历史积案，切实从源头上预防和化解矛盾纠纷。完善安全生产监管体制机制，认真开展重点领域专项整治，安全生产形势总体平稳。加强食品、药品安全监管，进一步规范和维护市场秩序。深化应急管理“一案三制”建设，突发公共事件应急处置能力不断提高。创建首都政治中心区防控机制，大力净化治安环境，社会秩序保持良好。

　　(四)加快推进改革开放，创新推动科学发展的体制机制

　　深化行政管理体制改革，崇文区与东城区、宣武区与西城区行政区划调整工作平稳有序，为落实城市总体规划、发挥核心区功能创造了有利条件。建立海淀区和昌平区产业统筹协调机制，推动大兴区和北京经济技术开发区融合发展。设立市级固定资产投资审批综合服务大厅，继续推进行政审批事项精简下放。深化国有企业改革，成功实施9个重组项目，退出劣势企业196家，国资监管体系进一步完善。深化金融改革创新，成立全国首家消费金融公司，争取多项国家金融创新试点，规范市区两级融资平台，新增要素市场9家。深化社会领域改革，制定深化医药卫生体制改革实施方案，成立首都医药卫生协调委员会，落实国家基本药物制度，实施药品统一集中采购。整合组建北京广播电视台，完成14家出版社转企改制。事业单位绩效工资改革稳步推进。

　　进一步扩大对外开放。开展跨境贸易人民币结算试点，推动首都机场口岸与天竺综合保税区实现大通关，扩大出口信用保险覆盖面，促进外贸出口恢复性增长，地区出口554.7亿美元、增长14.7%，规模接近国际金融危机前水平。健全服务贸易促进协调机制，落实服务外包政策，促进服务贸易快速发展。大力吸引跨国公司地区总部，新增涉外驻京代表机构654家，实际利用外资63.6亿美元。鼓励支持企业“走出去”，对外协议投资增长2.5倍。成功举办世界旅行旅游大会等国际活动，城市对外交往务实深入。

　　加强发展合作与对口支援。与14家中央单位签订战略合作协议，进一步完善合作机制和对接服务，推进一批重点产业基地和项目落地。整合组建市级领导机构，统筹对口支援和区域合作。先后与河北等省区市签订合作框架协议，区域合作广度和深度不断拓展。全面启动新一轮援疆工作，认真落实对口支援西藏拉萨、青海玉树等任务。累计投入资金70亿元，圆满完成支援什邡灾后恢复重建任务，获得各方面高度评价。

　　(五)加强精神文明和民主法制建设，不断提升城市文明程度

　　深入推进爱国主义教育，成功举办了纪念抗战胜利65周年系列活动。开展“做文明有礼的北京人”、“垃圾减量日”等主题实践活动，推出了春节文化论坛、端午文化节、重阳文化节等一批传统文化活动。加强对互联网、手机等新兴媒体的管理，大力整治网络低俗之风。认真做好民族、宗教、侨务和对台工作，积极推动妇女、儿童、老龄、残疾人事业健康发展。

　　稳步推进基层民主政治建设，着力完善社区居民自治机制，完成第八届村委会换届选举。加强法制宣传和教育，推进法律服务领域民生工程建设，顺利完成“五五”普法各项任务。加强国防和后备应急力量建设，创新与武警总队的合作机制，扎实做好“双拥共建”工作，驻京部队在首都建设发展中发挥了重要作用。

　　各位代表，一年来，市政府及组成部门认真执行市人大及其常委会的决议和决定，坚持重大事项报告制度，自觉接受人大法律监督和工作监督。主动向市政协通报工作，自觉接受政协民主监督，认真听取各民主党派、各人民团体和各界人士意见。办理全国和市人大代表议案、建议1549件，政协建议案、提案1380件。加强政府法制建设，提请市人大常委会审议地方性法规5项，制定修改政府规章5项。做好地方性法规、政府规章和规范性文件清理工作，进一步减少行政审批事项。加强和改进行政执法工作，发挥监察、审计、财政的监督作用，深入推行绩效管理和督查督办，有力推动了重大决策和重点工作的落实。投入财政资金237亿元，全面落实了58件重要实事。

　　各位代表，过去一年首都发展取得的成绩，是党中央、国务院和中共北京市委坚强领导，各方面高度关心、大力支持，全市人民迎难而上、团结奋斗的结果。在此，我代表市人民政府，向全市各族人民和来自全国各地的劳动者，向人大代表、政协委员，向各民主党派、各人民团体和各界人士，向中央各部门、各兄弟省区市，向驻京解放军和武警部队官兵，向所有关心支持首都建设的港澳台同胞、海外侨胞和国际友人，表示崇高的敬意和衷心的感谢！

　　我们清醒地认识到，全市经济社会发展中的困难和问题还不少。一些长期性、结构性矛盾尚未根本解决，又出现了一些不容忽视的新问题。经济运行面临复杂形势，稳定物价、房价的压力较大；房地产投资占比仍然偏高，投资结构亟待调整优化；机动车过快增长，缓解交通拥堵刻不容缓；涉及群众切身利益的一些问题还没有得到很好解决，特别是入园难、看病难等问题比较突出；资源、能源、环境约束更加凸显，节能减排面临新的压力和挑战。我们也清醒地认识到，政府工作中还存在缺点和不足，政府自身建设和服务管理创新还需要加大力度，一些工作落实还有差距，少数工作人员责任意识、服务意识不强，政府效能建设、勤政廉政建设需要进一步加强。我们要正视困难，正视问题，以对党和人民事业高度负责的态度，深入分析研究，采取更为积极有效的措施，切实加以解决。

　　二、2011年主要任务

　　2011年是中国共产党成立90周年，也是“十二五”时期的开局之年，做好2011年经济社会发展各项工作意义重大。当前，世界经济格局正在发生深刻复杂变化，我国仍处于重要战略机遇期，经济发展长期向好的趋势没有改变。首都发展面临着难得的机遇，也面对着严峻的挑战。党中央、国务院对北京发展寄予厚望，要求在加快转变经济发展方式上走在全国最前面。各级政府、各部门要认清形势，牢牢把握主题主线，坚定必胜信心，切实增强机遇意识、忧患意识、责任意识，切实增强工作的前瞻性、科学性和创造性，创新发展理念，创新发展模式，埋头苦干，攻坚克难，奋发有为，以更高的标准实施人文北京、科技北京、绿色北京战略，努力实现“十二五”时期的良好开局，推动建设中国特色世界城市迈出坚实步伐。

　　做好2011年政府工作，要全面贯彻落实党的十七大和十七届三中、四中、五中全会以及中央经济工作会议、市委十届八次全会、全市经济工作会议精神，深入贯彻落实科学发展观，以科学发展为主题，以加快转变经济发展方式为主线，着力建设创新驱动的发展格局，着力扩大内需，着力提高人民生活水平，着力推动文化繁荣发展，着力创新社会服务与管理，着力抓好城乡一体化建设，着力提高城市精细化管理和宜居健康水平，实现经济平稳较快发展和社会和谐稳定，以出色的成绩迎接中国共产党成立90周年。

　　综合各方面因素，全市经济社会发展的主要目标是：地区生产总值增长8%；城镇登记失业率控制在2.5%以内；城乡居民收入实际增长7%；居民消费价格涨幅控制在4%左右；地方财政一般预算收入增长9%；单位地区生产总值能耗和二氧化碳排放量均下降3.5%、水耗下降4%。

　　为实现上述目标，2011年要重点抓好以下几方面工作：

　　(一)保持首都经济平稳较快发展

　　牢牢把握重要战略机遇期，巩固首都经济平稳较快发展的良好局面，为改善民生、调整结构、提升服务奠定坚实基础。

　　着力促进消费稳定增长。充分发挥消费主导作用，深入挖掘首都市场的巨大潜力，培育新的消费热点，实现社会消费品零售额增长10%左右。一是增强居民消费能力。认真落实国家收入分配政策，增加对城镇低收入群体和农民的补贴，提高社会保障水平，促进城乡居民收入普遍较快增长。二是推动消费结构升级。扩大保障性住房供应规模，积极发展文化消费、老龄消费、健康消费、网络消费等新兴消费，加快推进电子商务示范工程，改造升级特色商业街区。三是开拓消费市场。继续抓好各类主题促销活动，认真做好家电下乡和以旧换新，搞好农村集贸市场、连锁超市、农产品批发市场和零售网点建设，提升新城商业发展水平，扩大农村消费。四是大力发展旅游消费。积极推进北京旅游中心等重大项目建设，提升传统旅游品质，培育工业、科技、生态等新兴旅游热点，以旅游、会展带动外来消费快速增长。五是全面推进商贸流通规范化、现代化、特色化、国际化发展，加快建设国际商贸中心。

　　深入调整优化投资结构。坚持投资适度增长与优化结构、提高质量效益并重，着力抓好重大项目建设，努力扩大产业投资，实现固定资产投资增长10%以上。一是切实用好政府投资。2011年市政府固定资产投资拟安排347亿元，重点保障在建、续建项目，优先支持结构调整、自主创新、公共设施、民生工程等领域。进一步完善融资体系，多渠道筹集资金，保障重点项目和产业发展的资金需求。二是积极扩大民间投资。出台促进民间投资的实施意见，鼓励引导民间资本进入基础产业和基础设施、市政公用事业、社会事业等领域。三是统筹促进产业投资。实施重大产业项目带动工程，完善股权投资体系，加强创业发展引导，鼓励企业加大研发产业化和技改投入，不断增强产业发展后劲。四是进一步调整房地产投资结构，加大政策性住房投资力度，优化土地一级开发。

　　加强价格调控和市场监管。认真履行经济调节、市场监管的职能，切实做好价格稳定工作。完善绿色通道政策，保障农产品货源供应，加强生活必需品政府储备，必要时实行价格临时干预。建立市场价格信息发布制度，有效引导社会预期。落实价格补贴政策，建立健全社会保障相关标准与物价上涨挂钩的联动机制，保障困难群众基本生活。认真落实食品安全行动纲要，构建食品安全长效机制。建立基本药物质量监管和供应储备保障体系，确保“三品一械”质量安全。健全市场体系和社会信用体系，强化价格监管，严肃查处恶意炒作、串通涨价、哄抬价格等不法行为，严厉打击虚假广告、制假售假，坚决维护市场秩序，为人民群众营造放心消费的市场环境。

　　(二)深入推进自主创新和产业结构调整

　　坚持把结构调整作为加快转变经济发展方式的主攻方向，深度调整产业内部结构，不断提高经济的整体素质与核心竞争力。

　　全力推动中关村示范区建设发展。认真落实中关村示范区条例，大力推进体制机制创新，加快建设具有全球影响力的科技创新中心和我国战略性新兴产业的重要策源地。建设首都创新资源平台，完善中关村科技创新和产业化促进中心的工作机制，推动中央与地方创新资源整合。深化先行先试改革，积极配合国家有关部委，抓紧落实科技成果处置权收益权下放等五方面政策，争取设立统一监管下的全国场外交易市场。建设国家科技金融创新中心。落实高端人才政策，深入实施海外人才聚集、高端领军人才聚集工程，加快建设中关村人才特区。进一步加大支持力度，统筹安排100亿元政府资金，做好联合审批和落地服务，全力推动科技成果产业化。全面实施“十百千工程”和“瞪羚计划”，支持创新型企业开拓市场、做强做大，加快培育一批拥有自主品牌的世界级企业。优化自主创新体系，积极对接国家科技重大专项和重大科技基础设施建设，健全企业创新激励机制，促进新型产业组织加快发展，努力突破关键核心技术。实施标准战略，推动标准创制。加强知识产权保护，培育发展科技中介组织，促进知识产权的市场化运用。

　　大力促进产业结构优化升级。制定实施培育发展战略性新兴产业的意见，加快发展新一代信息技术、新能源汽车、节能环保、高端装备制造、生物医药、新能源、新材料和航空航天等新兴产业。落实重点产业振兴规划和实施意见，加快北京现代三工厂、数字电视产业园、中核科技园、三一北京制造中心等重点项目建设，提升高技术产业和现代制造业发展水平。出台促进生产性服务业发展的实施意见，支持信息服务、商务服务和物流等重点产业发展，推进石景山国家服务业综合改革试点区建设。大力发展金融产业，支持金融街适度拓展和南北连片，促进商务中心区功能集聚，推进丽泽金融商务区建设，增强服务国家金融决策、管理、信息中心功能，加快打造具有国际影响力的金融中心城市。完善文化创意产业聚集区发展扶持政策，抓好中国动漫游戏城、中国乐谷、传媒产业走廊、通州文化主题公园、古北水镇等重点项目，提升文化创意产业的创新能力和集约化水平。积极发展家政服务业，促进生活性服务业加快发展。认真落实与中央单位签订的战略合作协议，加快推进合作产业基地建设，促进军民融合式发展。

　　深入推进节能减排。把二氧化碳、氮氧化物、氨氮减排作为约束性指标，确保各项指标达到国家要求。强化目标管理和考核，突出抓好工业、建筑、交通运输、公共机构等重点领域节能。严格执行建筑节能标准，大力发展绿色建筑，加快既有建筑节能改造。加大高效节能产品推广力度，进一步淘汰落后产能，完成800蒸吨燃煤锅炉清洁能源改造。建立老旧机动车更新淘汰长效机制，新增公交车提前实施第五阶段国家排放标准，继续抓好新能源公交、环卫车的示范应用。推行合同能源管理，促进节能服务业发展。制定再生资源回收产业发展规划，加强资源循环利用技术的研发应用，积极推动清洁生产，大力发展循环经济和环保产业，倡导绿色低碳生活方式和消费模式。

　　(三)切实保障和改善民生

　　坚持统筹兼顾、突出重点，努力办好惠民实事，促进基本公共服务均等化。

　　全面实施扩大就业战略。把促进充分就业作为经济社会发展的优先目标，强化政府责任，实现城镇新增就业36万人以上。重点做好高校毕业生、农村转移劳动力、城镇就业困难人员、退役军人的就业工作，促进大学生自主创业和充分就业，推动城镇促进就业政策向农村延伸，统一城乡就业援助制度。研究制定“三高”退出企业职工的分流安置政策，鼓励城乡劳动力实现“绿色就业”。健全职业培训制度，完善公共就业服务平台，推进充分就业村和社区的创建工作。加强劳动关系协调，积极推行工资集体协商，切实维护劳动者合法权益。

　　完善覆盖城乡居民的社会保障体系。推动“新农合”市级统筹，启动市级公费医疗与职工基本医疗保险并轨，加快构建城乡一体化医疗保障新格局。健全社会保障待遇调整机制，提高城乡居民基础养老金等各项待遇标准。积极拓展社保卡服务功能。完善社会救助政策，对特困人员实行住院押金减免和出院即时结算，研究设立慈善医疗救助基金。完善居民家庭收入核定的联动机制，促进社会救助与其他社会保障制度有效衔接。大力扶持社会化养老服务，积极发展社区养老餐桌和托老所，推进养老机构标准化建设。加强残疾人社会保障和服务体系建设，关心残疾人精神文化生活，切实保障残疾人权益，支持残疾人事业发展。

　　全力做好住房保障工作。多渠道增加资金投入，加大政策性住房建设收购力度，加快配套设施建设，切实增加实物供给量，确保新建和收购20万套，力争竣工10万套。大力发展公共租赁住房，鼓励社会资金参与，完善集体建设用地建设租赁住房的政策。积极推进老旧小区更新改造，按计划完成棚户区改造。严格落实房地产市场调控措施，保持土地供应平稳有序，继续增加住房有效供给。大力整顿房地产市场秩序，抑制投资投机需求，坚决遏制房价过快上涨。坚持政府保障和市场供给相结合，积极探索完善住房制度，逐步形成符合首都实际的保障性住房体系和商品房体系。

　　促进教育事业优先发展。落实中长期教育改革与发展规划纲要，启动实施国家教育体制改革试点和重点建设项目，深入推进素质教育。制定加快学前教育发展的意见，编制区县学前教育三年行动计划，加大幼儿园新建改建力度，加强幼教师资培养，新增入园名额2万个，规范幼儿园准入和收费管理。完成中小学校舍安全工程，深化教师交流、学区化管理等改革，推动优质教育资源向农村地区、薄弱学校配置，促进义务教育均衡发展。完善现代职业教育体系，鼓励支持民办教育健康发展。继续实施市属高校“三大工程”和三年基建规划，支持在京高校建设世界一流大学和高水平大学。

　　提升医疗卫生服务水平。深化医药卫生体制改革，组建市医院管理机构，积极开展公立医院改革试点，加快编制区域卫生规划和医疗机构设置规划，鼓励引导社会资本举办医疗机构。建立健全公共卫生项目管理规范及考核办法，认真做好重大传染病监测与防控，不断完善院前急救体系。健全基层卫生服务机构补偿机制，加强医护人员培养培训，推行家庭医生服务。完善基层医疗卫生机构基本药物目录，规范基本药物招标采购。支持“十病十药”产品研发生产，进一步提高中医药服务能力。强化公立医院管理，在全市三级医院推广双休日全天门诊，方便群众看病就医。

　　加快文化体育事业发展。大力发展公共文化事业，推动建立广覆盖、高效能的公共文化服务体系。深入实施文化精品工程，努力提供更多优秀文化作品。加快建设首都图书馆二期等标志性文化工程，完善城乡结合部等地区公共文化设施网络，探索建立基层文化志愿服务机构，深化文艺院团和文化单位内部机制改革。全面推进三网融合试点。大力发展哲学社会科学，促进文学艺术、广播影视、新闻出版事业繁荣发展。办好首届北京国际电影季。切实做好地下文物和非物质文化遗产保护工作，推进历史文化名城整体保护。落实全民健身计划，深入实施全民健康促进十年行动规划，广泛开展群众体育活动，不断提高广大市民的身体素质，加快建设健康城市。

　　(四)加大统筹城乡区域协调发展力度

　　坚持强农惠农政策力度不减，完善区县功能定位实现机制，加快构建城乡一体、多点支撑、均衡协调的发展格局。

　　深入推进农村改革发展。完成集体林权制度改革主体任务和1230个乡村集体经济产权制度改革，扶持培育农民专业合作社示范社。鼓励发展新型农村金融机构，支持涉农企业上市。认真做好农村土地确权登记颁证工作，严格土地管理，严肃查处违法用地。加强以农田水利为重点的农业基础设施建设，打造一批农业基础设施建设和综合开发核心示范区，推进国家现代农业科技城、农业生态谷等重点项目。完善都市型现代农业实现形式，促进农业生产经营专业化、标准化、规模化、集约化，积极发展农产品加工业和农村服务业。实施新一轮“菜篮子”工程，新建基本菜田5万亩，提高蔬菜自给率。多渠道增加农民收入，确保低收入农户稳定增收。认真做好世界草莓大会、种子大会和国际园林博览会筹备工作。

　　加快郊区城镇化步伐。基本完成城乡结合部城市化工程50个重点村的拆迁任务，统筹解决好产业发展、农民就业和社会保障等问题。遵循城市发展客观规律，扎实推进规划新城建设，增强对中心城区功能和人口疏解的吸引力，在发展新区加快打造地区生产总值500亿元级、1000亿元级的经济强区。研究制定促进农转居的政策措施，完善基础设施和产业发展规划，高标准建设一批示范小城镇和全国千强镇。加快社会主义新农村建设，健全“5+3”工程运行管护长效机制，继续开展农村抗震节能住房建设和既有农房保温节能改造。启动10个新型农村社区建设试点，推动城市服务管理向农村延伸。深入推进山区搬迁工作，确保农民“搬得出、稳得住、能致富”。

　　努力推动区域协调发展。充分发挥首都功能核心区行政区划调整的作用，坚持和完善各项政策，加快发展现代服务业和文化创意产业，扎实做好保护性修缮和对接安置工作，不断提高发展质量、增强服务功能。加快中关村科学城、未来科技城建设，着力打造北部研发服务和高新技术产业带。加大基础设施建设和重大产业项目布局力度，下更大决心聚焦通州，集中力量，力求取得重大突破；进一步做强临空经济区，着力发展高端商务、现代物流和先进制造业，提升东部地区发展水平。深入推进城南行动计划，完成一批重大基础设施项目和民生工程，认真抓好南中轴线规划建设，做好北京新机场开工建设准备工作，着力打造南部高技术制造业和战略性新兴产业发展带。出台加快西部地区转型发展意见，重点抓好新首钢高端产业综合服务区、永定河绿色生态发展带建设，积极推进燕房石化新材料、窦店现代制造业基地发展。大力支持怀柔文化科技高端产业新区、门头沟国家生态修复科技实验区、延庆国家绿色能源示范县、密云国际绿色休闲旅游产业综合示范区和平谷绿色能源基地建设，大力发展沟域经济，打造新型山区生态经济带。

　　(五)着力提高城市精细化服务管理水平

　　立足做好“四个服务”，坚持标本兼治、多措并举，破解城市服务管理难题，切实提高可持续发展水平。

　　综合治理交通拥堵。全面实施缓解交通拥堵各项措施，多管齐下、分阶段做好各项工作，确保见到实效。加大轨道交通建设力度，抓好在建线路建设，争取中心城两条新线分段开通。继续完善路网结构，实施快速路网和重点地区拥堵节点改扩建工程，推进微循环系统建设，开工建设东西二环等地下隧道。改善公共交通出行条件，增设向小区延伸的公交专线，建成阜石路大容量快速公交线路，新增一批公交专用道，公交出行比例达到42%。加快建设智能交通管理系统，优化重点区域交通组织，研究制定交通严重拥堵应急预案，完善高峰时段区域限行措施，依法严格管理，提高交通服务管理水平。实施小客车总量调控，保持全市各级党政机关公务用车零增长，引导机动车合理使用。优化自行车和行人出行环境，倡导绿色出行。

　　加强人口调控管理。认真落实中央关于“大城市要加强和改进人口管理”的要求，抓紧研究制定人口有序管理的一揽子政策措施。建立健全人口管理体制，严格落实目标责任制。发挥产业发展对人口规模的调节作用，制定行业准入标准，提升传统产业组织化程度和现代化水平。优化配置公共服务资源，推动中心城区功能疏解，引导人口合理分布。加强人防工程使用管理，规范房屋出租行为，推广农民出租房屋集中管理新模式，实现居住人口和出租房屋管理信息化。研究实施居住证制度，提高流动人口服务水平。加强与周边省区市协作，探索区域人口调控综合协调机制，促进人口有序流动。坚持计划生育基本国策，改善计划生育服务。切实保障妇女儿童权益，积极应对人口老龄化。

　　提升城市运行保障水平。围绕增能力、保供应，加强市政基础设施建设。实行最严格的水资源管理，推进南水北调市内配套工程建设，做好建设节水型社会试点工作，抓好永定河、北运河、潮白河三大水系综合治理，加强湿地保护，提升生态清洁小流域治理水平。完成东南热电中心建设，推进供热计量收费改革，研究制定采暖费“暗补变明补”和困难群体采暖保障措施。启动陕京天然气四线、大唐煤制气进京管线建设前期工作，加快远郊区县供气干线建设。提高城乡邮政服务水平。制定智慧城市行动纲要，积极推进物联网、云计算示范应用，加快城市信息化建设步伐。

　　加大环境建设力度。加快鲁家山等生活垃圾焚烧设施建设，继续治理非正规垃圾填埋场，在1200个小区、1200个村庄开展垃圾分类达标试点，大力推动建筑垃圾资源化利用。加快污水处理厂和管网建设改造，提高再生水利用能力。开展“精细管理、美化市容”活动，推进城六区重要交通干道两侧遗留项目整治，提升主要道路环境景观，抓好环境薄弱地区和农村环境整治。加快南海子等郊野公园、滨河森林公园以及两道绿化隔离带建设，改造山区低效林和平原防护林，实施城市增绿添彩工程，全市林木绿化率达到54%。全面落实清洁空气行动计划，力争二级和好于二级天数占比达到75%。

　　(六)进一步深化改革和扩大开放

　　坚持把改革开放作为加快转变经济发展方式的强大动力，下更大力气创新体制机制，扩大对内对外开放。

　　深入推进重点领域和关键环节改革。一是继续深化行政管理体制改革，进一步精简和规范行政审批事项，下放行政审批权限。完善投资审批大厅运行机制，强化审批后续监管。优化财政支出结构，统筹保障重点支出，加强审计监督。全面完成乡镇机构改革。二是深化国有企业公司制股份制改造，完善法人治理结构，加快兼并重组，支持市属企业整体上市或主业资产上市，继续推动劣势企业退出，提高国有经济产业集中度。三是落实促进中小企业发展的实施意见，进一步完善扶持政策，规范市场准入，强化金融服务，减轻企业负担，认真解决制约中小企业发展的共性问题。四是积极发展要素市场，加快资本市场建设，推动设立文化产权等交易所，逐步形成统一的人力资源市场。五是理顺资源价格形成机制，适时开展天然气价格、阶梯水价改革。

　　努力提高对外开放水平。坚持进口和出口并重，“引进来”和“走出去”并重，引资与引智相结合，加快转变外经贸发展方式。抓好出口产业基地建设，支持双自主产品扩大出口，鼓励企业培育出口品牌。完善促进服务贸易的政策体系，推动服务外包示范区建设，大力发展新兴服务贸易。落实国家进口促进政策，扩大先进技术设备、关键零部件、能源和原材料进口。加强投资促进工作，引导外资更多投向高端产业，更加注重引进技术、管理和人才，推动存量外资企业转型升级。积极吸引国内外企业总部落户北京，促进总部经济实体化经营。落实支持政策、简化审批手续，推进对外投资便利化。深化京港、京澳合作，全方位开展城市对外交往，积极申办有影响力的国际活动，不断提升首都国际化水平。

　　扎实做好对口支援和区域合作工作。认真落实中央关于对口支援的部署与政策，推进经济、干部、人才、教育和科技援疆，多方面、多渠道支援西藏发展，全面落实玉树援建协议。进一步拓宽与周边地区合作领域，研究编制区域合作发展规划，充分发挥首都城市的服务辐射功能，积极推动首都经济圈建设。

　　(七)全力维护首都和谐稳定

　　加强社会主义精神文明建设。认真办好纪念中国共产党成立90周年系列活动，大力弘扬社会主义核心价值观，深入开展“做文明有礼的北京人”主题实践活动，实施公共文明工程，培育和塑造北京精神。积极开展文明区县、文明村镇、文明单位、文明社区创建活动，加强网络文明建设，加强未成年人思想道德建设和大学生思想政治教育。充分发挥工会、共青团、妇联等人民团体的桥梁纽带作用，切实做好民族、宗教、外事、侨务、对台工作。深化国防教育，加强国防后备力量建设，深入开展双拥共建活动，认真落实优抚安置政策，积极帮助退役军人解决实际困难。

　　加强民主法制建设。制定贯彻落实国务院加强法治政府建设意见的实施规划，改进政府立法工作，完成年度行政立法任务。坚持依法科学民主决策，深入推进政务公开，增强决策透明度和公众参与度。健全基层自治组织和民主管理制度，保证广大群众依法直接行使民主权利。拓展和规范法律服务，构建律师管理与服务新机制。制定“六五”普法规划，加强法制宣传教育。严格规范、公正文明执法，加强行政复议工作，强化行政监督和问责，提高政府执行力和公信力，努力使首都依法行政工作走在全国前列。

　　深入推进社会服务管理创新。加快社区规范化建设，健全枢纽型社会组织工作体系，促进社会工作者专业化、职业化发展，努力扩大志愿者队伍。完善网格化社会管理模式，加快实现社会服务管理的信息化、精细化。推进1700个服务站标准化建设，改善农村社区基础设施。进一步扩大“一刻钟社区服务圈”试点，落实社区基本公共服务指导目录，完善政府购买社会公共服务机制。

　　切实维护社会安全稳定。坚持用群众工作统揽信访工作，严格落实维稳责任制，做好重大事项社会稳定风险评估，完善群众利益表达机制，深入推进社会矛盾化解。强化安全生产综合监管，严格落实企业主体责任，加强城市运行安全和生产安全保障，加大重点行业和领域的安全隐患排查治理力度，坚决遏制重特大事故发生。增强防灾减灾能力，强化应急资源统筹协调，提升突发事件综合防范水平。按照一流标准，加强警务、消防能力建设。完善社会治安防控体系，严厉打击各类刑事犯罪活动，努力营造社会安定、百姓安宁的良好局面。

　　三、关于《北京市国民经济和社会发展第十二个五年规划纲要(草案)》的说明

　　市委、市政府高度重视“十二五”规划编制工作，成立了专门领导小组，多次召开专题会议，对事关首都发展的重大问题进行研究决策。市人大常委会、市政协分别提出了专题建议，为规划编制提供了重要支撑。根据市委十届八次全会审议通过的《关于制定北京市国民经济和社会发展第十二个五年规划的建议》精神，在广泛听取各民主党派、工商联负责人、无党派人士、专家学者和广大市民意见的基础上，市政府制定了《北京市国民经济和社会发展第十二个五年规划纲要(草案)》，已提请大会审议。

　　《纲要(草案)》共九篇，第一篇是新时期的战略选择，总结了“十一五”时期首都经济社会发展成就，明确了未来五年的发展目标；第二篇到第八篇，是“十二五”时期经济社会发展的主要任务，包括创新驱动发展、发展惠及人民、文化彰显魅力、城市服务生活、绿色塑造未来、改革激发活力、开放实现共赢等七个方面；第九篇是未来五年的行动路径。下面，我就几个问题作简要说明。

　　(一)“十一五”时期首都经济社会发展的主要情况

　　“十一五”时期是北京发展史上极不平凡的五年。五年来，在党中央、国务院和中共北京市委的坚强领导下，在全国人民的大力支持下，成功举办了一届无与伦比的奥运盛会，圆满完成了新中国成立60周年庆祝活动筹办任务，全面实现了“新北京、新奥运”战略构想，首都经济社会发展取得了历史性成就。

　　——经济发展迈上新台阶。与2005年相比，六项主要经济指标实现或大体实现翻番：经济总量由6970亿元增加到1.3万亿元以上，人均地区生产总值由5615美元提高到10000美元以上，地方财政一般预算收入由919.2亿元增加到2353.9亿元，社会消费品零售额由2912亿元增加到6200亿元以上，全社会固定资产投资由2827亿元增加到近5500亿元，地区进出口总额由1255亿美元提高到3014亿美元，首都经济综合实力显著增强。

　　——人民生活持续改善。“十一五”期间，城乡居民收入年均实际增长超过8%，教育、医疗、社保等财政支出分别比“十五”期间增长了1.8倍、1.7倍和1.6倍，就业服务和社会保障制度实现全覆盖，社会保障各项待遇标准均提高40%以上，城镇登记失业率控制在2%以内，社会事业发展继续走在全国前列。

　　——城乡面貌焕然一新。轨道交通运营里程由114公里增加到336公里，“区区通高速”目标全面实现。空气质量二级和好于二级天数占比提高了14.3个百分点，林木绿化率达到53%，单位地区生产总值能耗、水耗提前一年实现“十一五”目标，节能减排走在全国前列。率先实施区县功能定位，扎实推进新农村建设，城乡区域统筹发展的水平进一步提高。

　　实践证明，过去的五年是首都经济社会发展质量最高、综合实力提升最快、城乡面貌变化最大、人民得到实惠最多的时期之一。这些巨大的成就，极大地增强了北京作为全国政治中心、文化中心和国际交往中心的服务功能，极大地提高了首都的影响力和竞争力，极大地激发了广大群众推动科学发展的积极性和创造性，标志着首都发展进入了一个新的历史阶段。

　　总结“十一五”成就的同时，也要清醒地看到，前进的道路上还存在许多矛盾和问题，特别是一些不平衡、不协调、不可持续的问题需要高度关注。主要是：首都经济总体实力、竞争力和可持续发展能力需要进一步提高，三次产业内部结构还需要深度调整优化，加快转变经济发展方式任重道远；科技创新能力还不够强，对首都经济带动能力还需要进一步提高；城乡区域发展还不平衡，基本公共服务均等化水平需要进一步提高；城市建设管理中面临着人口、交通等问题，加强城市精细化管理任务仍然繁重；社会服务管理工作需要进一步加强，促进社会和谐还需要下更大力气。这些问题都需要我们努力加以解决。

　　(二)“十二五”时期首都经济社会发展的指导思想和主要目标

　　“十二五”时期是推动首都科学发展的关键时期。《纲要(草案)》全面贯彻落实科学发展观，体现了市委《建议》提出的指导思想，这就是：高举中国特色社会主义伟大旗帜，以邓小平理论和“三个代表”重要思想为指导，深入贯彻落实科学发展观，认真贯彻中央对北京市工作的一系列重要指示精神，以科学发展为主题，以加快转变经济发展方式为主线，顺应人民群众过上更好生活新期待，深化改革开放，全力推动人文北京、科技北京、绿色北京战略，进一步提高“四个服务”水平，努力打造国际活动聚集之都、世界高端企业总部聚集之都、世界高端人才聚集之都、中国特色社会主义先进文化之都、和谐宜居之都，推动北京向中国特色世界城市迈出坚实的步伐。

　　根据市委《建议》，《纲要(草案)》提出了“十二五”时期全市经济社会发展的主要目标。

　　——经济平稳较快发展。率先形成创新驱动的发展格局。地区生产总值年均增长8%，地方财政一般预算收入年均增长9%。中关村国家自主创新示范区初步建成具有全球影响力的科技创新中心，战略性新兴产业的支柱地位初步形成，服务业比重达到78%以上，“北京服务”、“北京创造”的品牌影响力明显增强。

　　——居民收入较快增加。城乡居民收入扣除价格因素年均增长8%，低收入者收入明显增加，中等收入群体持续扩大，收入差距不断缩小。市民健康水平得到显著提升，市民生活质量不断提高。

　　——城乡环境更加宜居。率先形成城乡经济社会发展一体化新格局。交通拥堵得到有效治理。林木绿化率提高到57%。单位地区生产总值能耗和二氧化碳、主要污染物排放持续下降。空气质量二级和好于二级天数占比达到80%。

　　——社会发展和谐稳定。基本公共服务均等化程度明显提高。城镇登记失业率控制在3.5%以内。城乡社会保障体系基本健全。中低收入群众的住房条件得到明显改善。社会服务和管理体制更加完善。

　　——文化大发展大繁荣。社会主义核心价值体系建设更加深入，市民文明素质和城市文明程度进一步提高。首都科技、教育、文化等资源优势充分彰显，城市文化软实力显著提升，全国文化中心功能显著增强。

　　——改革开放深入推进。有利于推动科学发展的体制与制度进一步健全完善，行政管理体制改革不断深化，行政效能进一步提高。对外开放的深度和广度进一步拓展，成为国际社会重大活动的重要目标地和国际一流的旅游中心城市。

　　这里，我就其中两个方面的重要目标作简要说明。

　　一是关于经济增速。提出地区生产总值年均增长8%，比“十一五”规划的目标低1个百分点，主要是考虑为加快转变经济发展方式创造良好环境，引导各方面把注意力放在提高发展质量和效益上。从首都经济的现实基础和增长潜力看，这个目标经过努力是可以实现的。

　　二是关于居民收入。城乡居民收入年均增长目标为8%，体现了居民收入增长和经济发展同步的要求。至于2011年城乡居民收入增长目标确定为7%，比“十二五”目标低1个百分点，比“十一五”目标高1个百分点，主要是考虑当前价格形势，以及收入增长提速的客观过程。

　　需要进一步说明的是，《纲要(草案)》还提出了“两个率先”的具体目标，就是率先形成创新驱动的发展格局、率先形成城乡经济社会发展一体化新格局。这些奋斗目标，描绘了未来五年首都现代化建设的宏伟蓝图，符合中央的要求和人民群众的期待，既鼓舞人心又艰巨繁重。我们要全力以赴、真抓实干，确保各项目标任务如期实现，为建设中国特色世界城市奠定坚实基础。

　　(三)“十二五”时期经济社会发展的主要任务

　　《纲要(草案)》对“十二五”时期的经济建设、社会发展、城市建设、改革开放等各方面工作进行了全面部署，提出了明确的任务和政策措施。

　　1.关于自主创新和结构调整。《纲要(草案)》把自主创新和结构调整作为今后五年的首要任务，强调要努力推动首都经济走上高端引领、创新驱动、绿色发展的轨道。一是建设国家创新中心。关键是要抓好中关村示范区建设，着力搭建首都创新资源平台，完善创新激励和支持机制，推进创新成果产业化，强化教育战略支撑作用。二是提升经济发展质量。要扩大首都经济优势，深度推进产业升级，统筹经济发展政策。特别是要整体塑造“北京服务”、“北京创造”品牌，增强产业竞争力和影响力。三是构建战略发展高地。着眼于城市发展空间战略调整和功能优化配置，加快形成“两城两带、六高四新”的发展格局。六大高端产业功能区增加值占全市经济比重力争达到50%。

　　2.关于保障和改善民生。《纲要(草案)》提出，要更加注重统筹经济社会协调发展，全面提升社会服务管理水平，使发展成果更好地惠及人民。一是扩展优质多样的公共服务。继续坚持积极的就业政策导向，为劳动者创造充分就业机会。建立城乡一体化的社会保障体系，稳步提高保障待遇水平。大力发展各项社会事业，办好人民满意的教育，建设健康城市。加大政策性住房建设力度，全面实现广大市民住有所居。二是建设公正和谐稳定的社会。完善充满关爱的社会福利网络，实现社会福利制度向适度普惠型转变。深入推进社会服务管理创新，积极化解社会矛盾，强化公共安全管理和社会治安综合治理，加强社会主义民主法制建设。三是构建适应首都发展的人口格局。合理调控人口规模，改进人口服务管理，引导人口合理布局。

　　3.关于首都文化建设。《纲要(草案)》把文化软实力建设放在更加突出的位置，作了全面部署，明确提出要树立“大文化”发展理念，充分发挥首都文化、科技、教育等优势，努力打造中国特色社会主义先进文化之都。一是整体保护历史文化名城。要重构历史文化魅力走廊，推进历史名城风貌保护，让文化元素融入城镇乡村。二是提供丰富多彩的文化服务。着力提升公共文化服务，推进文化产品创新，建设共有精神家园。三是提升文化创意产业竞争力。重点是打造文化服务功能区，推动文化创意产业升级，优化发展环境，巩固文化创意产业的支柱地位。四是推动文化传播的创新拓展。要扩大文化产品和服务输出，构建高效的文化传播网络，鼓励优秀文化走向世界，显著提高首都文化的国际影响力。

　　4.关于城乡建设和服务管理。《纲要(草案)》着眼于城市服务生活，从三个方面提出了要求：一是构筑城乡区域协调发展格局。要深化区县功能定位，引导市域按功能分类发展，振兴城市发展的薄弱地区，建设功能完善的现代新城，发展各具特色的城镇乡村。二是建设系统完善的基础设施。要着力抓好资源能源供应，保障安全可靠供水，加快能源结构调整，加强信息通信高速网络和枢纽建设。特别是集中精力抓好交通拥堵治理，大力落实公交优先战略，加快中心城轨道交通建设，努力实现便捷出行。三是构建精细智能的城市管理。坚持规划、建设、运行、管理、服务的统筹协调，保障城市安全协调运行，提高抗灾应急能力，促进城市服务管理精细化、智能化。

　　5.关于生态文明建设。这是实现全面建设小康社会奋斗目标的新要求。《纲要(草案)》明确提出了污染治理、绿化美化、节能减排和应对气候变化的目标任务。一是营造清新城市环境。继续改善大气环境质量，实现垃圾全处理，全面治理水污染，有效控制噪声等其他污染。二是切实加强生态建设。扩大城市森林和绿地面积，提高山区森林质量，全面改善城市河湖水环境。三是共建宜居绿色家园。深入推进节能降耗，倡导绿色生产生活，积极应对气候变化，进一步提高首都生态文明水平和可持续发展能力。

　　6.关于改革开放。《纲要(草案)》强调，要围绕加快转变经济发展方式，着力推进重点领域和关键环节改革，不断完善社会主义市场经济体制。要深化行政管理体制改革，增强市场主体活力，完善现代市场体系，深化社会领域改革，推进农村体制改革，加快构建有利于科学发展的体制机制。要从首都功能定位出发，统筹对外开放和区域合作。加强国内经济合作与交流，推动形成首都经济圈。积极参与全球经济分工，吸引聚集高端企业总部，建设国际商贸中心，拓展利用外资和境外投资。强化首都国际交往中心功能，不断扩大国际交流合作，努力提升城市国际影响力。

　　各位代表！全面完成2011年和“十二五”时期的任务，对政府工作提出了新的更高要求。我们一定要坚决执行市人大及其常委会的决议和决定，自觉接受人大法律监督和工作监督。认真听取各民主党派、工商联、各人民团体和各界人士的意见建议，自觉接受政协民主监督。全面正确履行政府职能，深入推进依法行政，创造性地开展工作，大力加强政府自身建设，不辜负党和人民的重托。

　　我们要不断深化行政管理体制改革。继续精简、规范行政审批事项，切实转变政府管理经济的方式，发挥市场配置资源的基础性作用，加强市场监管、社会管理和公共服务职能，加快建设服务型政府和法治政府。加强公务员教育和管理，深入实施绩效管理，加大政务公开力度，不断提高行政效能和服务水平。

　　我们要切实做好新形势下群众工作。牢固树立群众观点，注意把握群众诉求，统筹协调好各方面的利益关系。建立健全开展群众工作长效机制，提高群众工作的针对性和实效性，努力维护社会和谐稳定。

　　我们要深入开展廉政建设和反腐败斗争。认真贯彻落实十七届中央纪委六次全会精神，坚持以人为本、执政为民，严格执行党风廉政建设责任制，认真遵守廉政准则，深入推进廉政风险防范管理，加强对行政权力的监督制约，加大违纪违法案件查办力度，着力解决发生在群众身边的腐败问题，切实做到清正廉洁、务实为民。

　　各位代表！首都北京迈上了新的历史征程，美好前景催人奋进。让我们更加紧密地团结在以胡锦涛同志为总书记的党中央周围，高举中国特色社会主义伟大旗帜，以邓小平理论和“三个代表”重要思想为指导，深入贯彻落实科学发展观，在中共北京市委坚强领导下，解放思想，抓住机遇，开拓创新，以更大决心和勇气全面推进首都现代化建设各项事业，在中国特色社会主义道路上继续阔步前进！
